# Ultralow amounts of DNA from long-term archived serum samples produce high-quality methylomes

**DOI:** 10.1186/s13148-021-01097-3

**Published:** 2021-05-12

**Authors:** Marcin W. Wojewodzic, Magnus Leithaug, Marianne Lauritzen, Robert Lyle, Sofia Haglund, Carl-Johan Rubin, Philip A. Ewels, Tom Grotmol, Trine B. Rounge

**Affiliations:** 1 Department of Research, Cancer Registry of Norway, Oslo, Norway; 2 Department of Medical Genetics, Oslo University Hospital and University of Oslo, Oslo, Norway; 3 Centre for Fertility and Health, Norwegian Institute of Public Health, Oslo, Norway; 4 Science for Life Laboratory (SciLifeLab), Department of Biosciences and Nutrition, Karolinska Institute, Huddinge, Sweden; 5 Department of Medical Biochemistry and Microbiology, Uppsala University, 75123 Uppsala, Sweden; 6 Science for Life Laboratory (SciLifeLab), Department of Biochemistry and Biophysics, Stockholm University, Stockholm, Sweden; 7 Centre for Bioinformatics, University of Oslo, Oslo, Norway

**Keywords:** Biobanks, Epigenetics, Methods, Methylome, Serum, Storage, WGBS

## Abstract

**Background:**

Long-term stored serum is considered challenging for epigenomic analyses: as there are no cells, circulating DNA is scarce, and amplification removes epigenetic signals. Additionally, pre-analytical treatments and storage might introduce biases and fragmentation to the DNA. In particular, starting with low-input DNA can result in low-diversity libraries. However, successful whole-genome bisulphite sequencing (WGBS) of such serum samples has the potential to open biobanks for epigenetic analyses and deliver novel prediagnostic biomarkers. Here, we perform WGBS using the Accel-NGS library preparation kit on ultralow amounts of DNA from long-term archived samples with diverse pretreatments from the Janus Serum Bank.

**Results:**

Ninety-four of the 96 samples produced satisfactory methylation calls; an average of 578 M reads per sample generated a mean coverage of 17× and mean duplication level of 35%. Failed samples were related to poor bisulphite conversion rather than to sequencing or library preparation. We demonstrate the feasibility of WGBS on ultralow DNA yields from serum samples stored up to 48 years.

**Conclusions:**

Our results show the potential of large serum biobank collections for future epigenomic studies and biomarker discovery.

**Supplementary Information:**

The online version contains supplementary material available at 10.1186/s13148-021-01097-3.

## Background

Epigenome-wide association studies (EWAS) have identified associations between numerous epigenetic regions and corresponding phenotypes, including diseases [1]. The availability of high-quality DNA and cost-effective methylation arrays has been essential to these discoveries. Epigenetics has traditionally been performed on samples with large amounts of DNA such as whole blood or other tissues, producing accurate results. The lack of white blood cells has previously considered serum samples as difficult for EWAS. However, serum samples may include ultralow amounts of circulating DNA [2]. Moreover, it has recently been shown that GWAS can be successfully performed on such serum samples [2] which gives rise to the question about the feasibility of performing WGBS on this material. It has been shown that sample storage has negligible effects on methylation measurements in the case of whole blood [3]. However, different sample processing can be applied for serum samples and it is not known if this would have an effect on methylation.

With the Infinium Methylation EPIC array, researchers can interrogate up to 850 k methylation sites across the genome, and this constitutes only 3% of the total number of CpGs, biased towards gene, promoter and enhancer regions. Among the remaining 97% of unexplored CpGs, future biomarkers discoveries are possible and can be interrogated using sequencing approaches. The downside of the array platforms is their high DNA requirement, which hampers use of the archived serum samples and directs the attention of the scientific community towards sequencing.

The main aim of this study was to evaluate the potential for use of large collections of serum samples for epigenomic research. To this end, we performed, for the first time, a whole-genome bisulphite sequencing (WGBS) pilot experiment on 96 samples from the Janus Serum Bank in Norway [4]. Specifically, we addressed whether ultralow amounts of DNA extracted from long-term archived serum samples allowed for high-quality CpG methylation calls. A subsidiary aim was to assess the duplication level, a potential problem for low-quantity DNA material from such samples.

## Results

### DNA yield

**Fig. 1:**
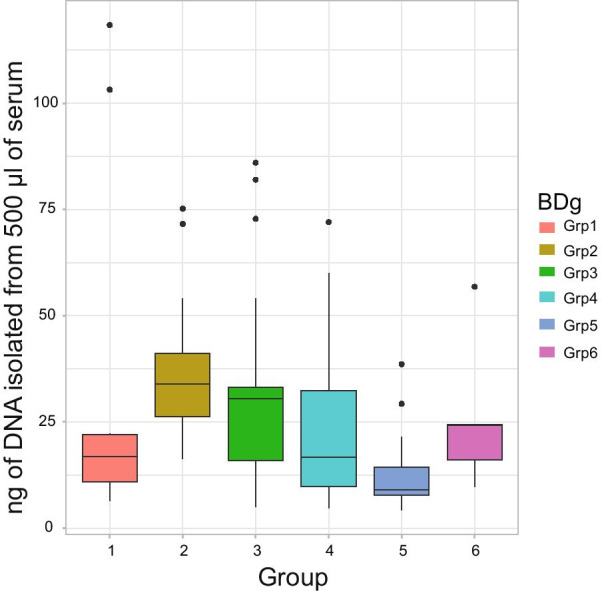
Yield of DNA isolated from 500 µl of serum for each group and quantified by Qubit. Box plot with median marked

The DNA yields obtained from the serum samples of all blood donor groups were substantially lower than the manufacturer’s Epic array protocol recommendations (~ 10% of recommended yield as measured by Qubit), thus disqualifying all samples from methylation arrays and justifying the use of a next generation sequencing strategy. Quantified DNA gave a median amount of 22.5 ng/500 µl of serum (sd 49.0 ng, Fig. 1) which was sufficient for performing the WGBS using the Accel library kit. One sample had an extremely high yield of DNA after isolation, compared to the rest of the samples (456 ng), and therefore, it was excluded from statistical analysis comparing DNA yield between groups. We found statistically significant differences between the yield of DNA obtained for different groups (ANOVA, F_5,87_ = 4.25, p = 0.0016). Group 5, which had no additives, had a lower yield compared to Group 1 (p = 0.039). We successfully prepared libraries from DNA amount ranging from 4 to 20 ng as starting material. Although traces of the extracted DNA from the samples on TapeStation showed different distributions of DNA fragments, shearing treatment in the Covaris instrument brought the distribution of the samples to a similar range (data not shown). All 96 samples were suitable for bisulphite treatment. The library preparation resulted in a uniform library size of ~ 300 bp visible on TapeStation (data not shown).

### Sequencing of the libraries

We gradually decreased the PhiX concentration during individual sequencing runs from 25 to 12%. While doing this, we did not record any substantial decrease in either the quality or yield of the sequencing. The use of different percentages of PhiX DNA spiking during sequencing did not have any effect on the overall sequencing yield for the serum samples (p = 0.14), suggesting 12% spiking with PhiX for future projects as a safe and cost-effective value for NovaSeq 6000. We produced a total of 110 916 M reads.

### DNA input change over time

To estimate potential degradation of DNA samples over time, we used evenness of sequencing coverage and GC content. The calculated evenness coefficient never surpassed the critical value of 2.5 [5] with average mean 1.04 (range 0.73–1.68). We tested differences between groups using a statistical model. In the model, we used storage time of the samples as an ordered factorial explanatory variable and the sampling procedures were included as a covariate while the evenness coefficient was the response variable (ANOVA, F = 0.47, *p* = 0.75 and F = 1.80, *p* = 0.18 for the time and sampling procedure, respectively). Further, we used GC content to assess whether degradation of the DNA has occurred over the time the samples were in storage. The mean GC content of a sequenced library was 21.6% (range 21–23%), suggesting no obvious degradation of DNA caused by storage time or the temperature at which the samples were stored. We investigated and excluded this possible degradation further with a statistical model. In the model, we used storage time as an ordered factorial explanatory variable and the sampling procedures were included as a covariate while the GC fraction was the response variable (ANOVA, F = 0.95, *p* = 0.44 and F = 1.37, *p* = 0.24 for the time and sampling procedure, respectively).

### General sequencing statistics

**Fig. 2:**
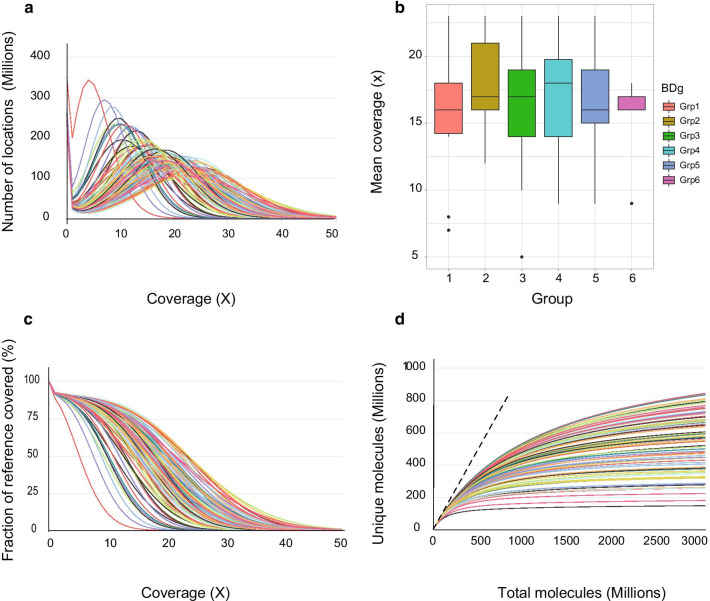
Coverage and duplication information in the sequencing project for the 96 samples. **a** Distribution of the number of locations in the reference genome with a given depth of coverage. *X* axis is the coverage, and *y*-axis is the number of locations (millions). **b** Mean coverage box plot per sampling procedure. Median marked. **c** Fraction of the genome covered by at least *X* reads. *X* axis is the coverage, and Y axis is the fraction of the reference genome. **d** Complexity curves, total molecules vs unique molecules. The data up to *x* = 600 M are based on actual results, beyond that is a simulation/projection showing the expected diminishing returns of additional sequencing in terms of unique reads

**Fig. 3:**
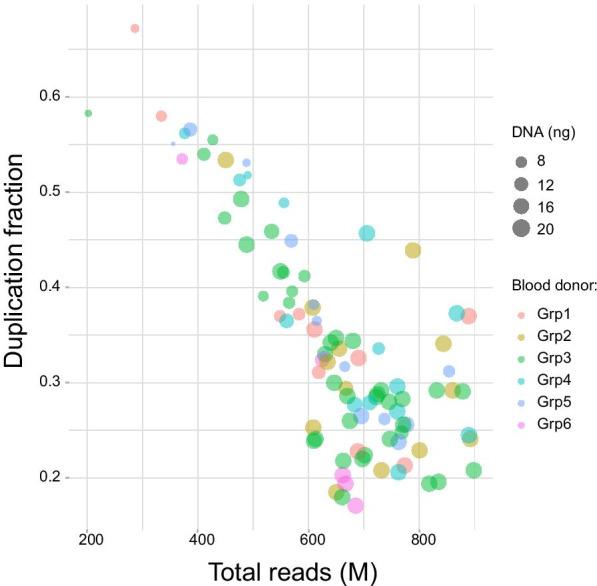
Duplication rate as a total of reads produced for each sample. *X* axis is the total number of reads produced, and *Y* axis shows the proportion of duplicated reads. The amount of the DNA (ng) entering library preparation was labelled with different sizes of the circles and group by colours

The mean insert size was 158 bp (range 149–176 bp), and the mean coverage was 17× (5×–23×). The mean fraction of the genome that was covered at least 10× was 76% (12–87%), and the mean fraction covered at least 1× was 92% (89–93%) (Fig. 2). The mean duplication rate was 34% (17–67%) and this was strongly correlated to the sequencing depth of the sample (ANOVA test, F = 180.7, *p* << 0.001), DNA concentration prior to the bisulphite treatment (F = 12.1, *p* < 0.0079), and to the sampling procedure (F = 2.4, *p* = 0.041, Fig. 3). Mean total number of reads was 645 M (202–898 M). We recorded a mean of 83.3% of methylated Cs in CpG context (80.4–84.8%) and 0.53% methylated Cs in non-CpG context (0.2–9.5%). Two samples had methylation of non-CpG context exceeding 2% and were thus excluded from downstream analysis (Additional file 1: SFig 1). DNA amounts used for these samples were 6 and 13 ng and sequencing from these two samples resulted in 515 M and 601 M reads produced with 45% and 27% duplications, respectively, which may suggest a random effect of bisulphite conversion.

### Trade-offs between DNA input, sequencing depth and duplication rate

The amounts of DNA (ng) for library preparation were between 4.14 and 20 ng of DNA. Around 60% of the samples were in the high range with 40% of samples below 20 ng representing typical DNA yields found in Janus samples. We found that after an average of 600 M reads, we had depleted the possible information gain from additional sequencing (Fig. 2d) and that we could not produce any more cost-effective information from any of the samples.

The sequencing identified a large number of unique cytosines (Cs). However, the number of Cs detected in each sample strongly depended on sequencing depth. We found no significant differences between the groups (ANOVA, F_5,88_ = 0.89, *p* = 0.487) with mean of 8620 measured Cs (range 8059–9586).

**Fig. 4:**
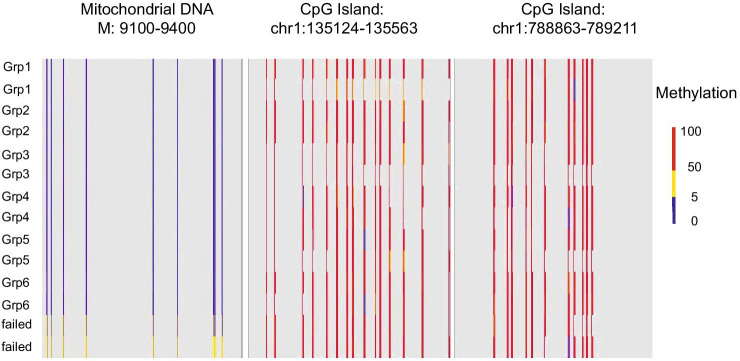
Methylation in mitochondrial DNA and two random CpG Island locations for 12 randomly chosen samples, representing all sample groups (1–6) and two failed samples. The bedGraphs represent the methylation values at given genomic locations. Scale represents methylation value

There was no relationship between the input DNA amount used for library preparation and methylation call rates for the samples that passed quality control for any of the sample protocols used (*p* = 0.63 and *p* = 0.16, for DNA input and blood donor groups, respectively). Methylation conversion rate was good in our studies as suggested by the low non-CpG context methylation with mean 0.33% (sd = 0.032), that was independent of the group (ANOVA, F_5,88_ = 0.75, *p* = 0.58) and unmethylated CpGs visible in the mitochondrial DNA fraction of the genomes (Fig. 4). There was no significant difference between methylation in CpG context between groups (ANOVA, F_5,87_ = 1.06, *p* = 0.39).

### Estimation of cell composition using two existing approaches

**Fig. 5:**
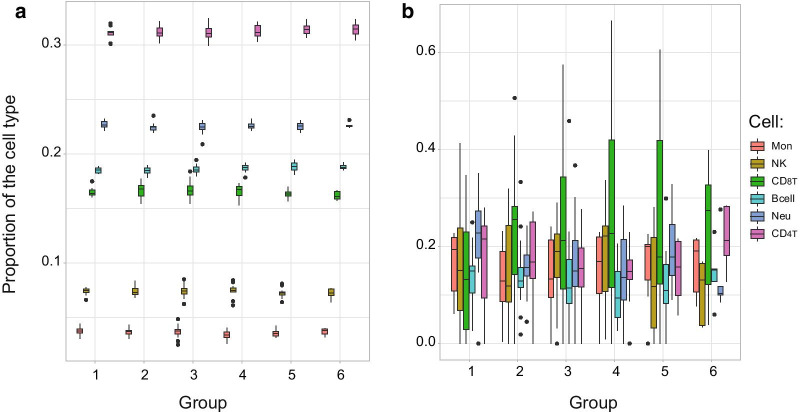
Mean cell composition estimated from methylation signatures for **a** Houseman and **b** methylCC method. *X*-axis is the blood collection group (1–6) and cell type, and *Y*-axis shows the proportion of cell types

MethylCC and Houseman methods were used for calculating cell composition on these samples. We have recorded differences in different cell populations between these two approaches. For the Houseman method, we observed B cell population 18.7% (sd = 0.159), CD4 + T cells 31.2% (sd = 0.160), CD8 + T cells 16.5% (sd = 0.201), monocytes 3.6% (sd = 0.138), neutrophils 22.6% (sd = 0.101) and natural killer cells 7.4% (sd = 0.118) (Fig. 5A). MethylCC produced more variable results. We observed B cell population 13.0% (sd = 1.80), CD4 + T population 16.9% (sd = 2.74), CD8 + T 22.7 (sd = 4.46), monocytes 15.4% (sd = 1.17), neutrophils 16.8% (sd = 3.02) and natural killer cells 15.2% (sd = 2.86) (Figs. 5b).

## Discussion

### Methylome sequencing is possible from serum biobanks

Prediagnostic serum biobanks are important resources for understanding disease development. The Janus Serum Bank also contains detailed metadata including sampling regimes throughout the collection time, clinical data and traits from health surveys. In this context, information that can be assembled from DNA sequences, such as methylation, is of high value. While epigenetic studies require suitable samples to analyse, large serum collections remain unexplored as they were assumed to provide insufficient amounts of DNA for methylation analysis. Moreover, the methylome analyses from such biobanks were considered as high-risk and unfeasible projects.

In our study, we have demonstrated that 500 µl archived serum yields DNA quality and quantity sufficient to produce high-quality methylome profiles using next-generation bisulphite sequencing. Methylomes can be linked to health conditions and other important covariates, such as age or smoking habits, in order to answer vital scientific questions.

### Acrylamide should be used during DNA extraction from stored serum samples

First, we have shown that acrylamide (a DNA carrier used during precipitation step in isolation procedures) improved DNA yield from these samples, compared to previous published data using the same biobank samples. The median content of isolated DNA from the 96 Janus Samples was 22.1 ng/500 µl compared to the previously reported value of 15 ng/500 µl for other samples from the same biobank [2]; this was statistically significant (*p* = 0.012). Our results support previous studies using Janus samples that have shown that the differences in yield of DNA between blood donor groups could be attributed to the pre-processing of the serum samples [2]. Groups 2 and 5 have very different DNA levels despite neither one having any additives during collection, suggesting that procedures at ‘health survey samples’ versus ‘red cross blood donor samples’ introduced an unknown factor, probably during sampling procedure, that contributed to this difference.

### Accel kit produced quality libraries

We have used the Accel kit that was designed for ultralow DNA concentrations. We successfully created all individual libraries, using a range of DNA concentrations. Forty percent of the samples had DNA inputs below 20 ng that represents a realistic range of DNA yields obtained from Janus samples and other serum collections.

### Decreasing PhiX does not compromise quality of libraries

WGBS requires additional spiking with PhiX to increase diversity of the library; however, this addition increases costs per sample. We have optimized the PhiX concentration with a gradual decrease in spiking from 25 to 12% on the NovaSeq platform. We found no difference in quality or yield related to PhiX ratio, and thus, we propose a 12% spike for future methylomes projects. However, a further decrease in PhiX might be possible. NovaSeq 6000 is a cost-effective instrument for future methylation projects.

### DNA extracted from old serum samples is stable

There was no bias in evenness of coverage across the samples nor any bias due to GC context, across 96 sample sequences, indicating that long term-storage samples can be used in epigenomic studies. Evenness of coverage was calculated as the coefficient of variation as previously described [5] and can point to no systematic loss of genomic regions. The GC content suggests that no obvious degradation of DNA was caused by storage time or the temperature (− 25 °C) at which the samples were stored and also that no contamination with other DNA sources and no problems with library preparations. The stability of the DNA could also be indicated by previous GWAS feasibility studies from the Janus Serum Bank [6]. Also, methylation marks were shown to be stable up to 17 years in blood samples [3]. Our study further confirms that samples can be stored and assessed for methylation. These findings were robust across different pre-analytical treatments and preservation times as is often the case in long-term biobank archives.

We observed failed bisulphite conversion in 2 out of 96 samples. This was detected by unexpectedly high levels of methylation in non-CpG context and higher methylation values in mitochondrial DNA for these two samples, compared to the rest. These did not have lower input amounts of DNA nor lower complexity compared to the successful samples. The failed samples were likely related to problems with bisulphite conversion rather than with sequencing or library preparation. In normal samples, mitochondrial DNA lacks methylation, so increased methylation in this genomic region, combined with the high non-CpG methylation, suggests that there was incomplete bisulphite conversion leading to false methylation calls across all cytosines. These signals are technical artefacts due to the incomplete conversion that disqualifies these two samples.

### Trade-off between duplication rate and DNA input

The mean duplication rate of 34% (17–67%) was quite high, driven primarily by low DNA concentration prior to the bisulphite treatment and to a lesser extent by the sequencing depth, and the sampling procedure. This suggests that diversity of the library can be compromised by insufficient quantities of DNA entering the reaction. This is also not surprising as bisulphite treatment is a harsh procedure that can degrade up to 90% of the molecules [7].

### There is a need to improve deconvolution methods from WGBS methylome

A major challenge in the downstream analysis of DNA methylation data is the variability introduced by intra-sample cellular heterogeneity. An example is whole blood, which is a mixture of cells with distinct DNA methylation profiles. This source of variability can compromise results and has to be taken into account. Current methods to estimate the cell type proportions are most appropriate for array technology and with a known reference composition such as whole blood [8]. Adjusting for the cell populations has been also shown in this study pivotal for epigenetics analysis. In our study, cell composition was inferred from the methylation data, assuming the expected fraction of the circulating DNA from the serum sample originates from blood cells. This conservative approach assumes that all DNA originate from blood related cells, while cell-free floating DNA present in the blood can also come from other tissues [9]. However, if the seen variability is a part of phenotype, we risk of removing signal. We found differences between methylCC and Houseman methods in estimating the cell type proportions. Our results therefore suggest an urgent need for the community to improve these methods for serum data. MethylCC is a new, untested approach that uses sequencing data and can introduce unknown biases, so it should be used with care. Although the reference is done on blood cell types, one can expect debris of the cell components from the blood in the serum samples, as well as cell-free DNA from other cells that these algorithms do not account for.

## Conclusion

To our knowledge, we present here a novel study to show that archived serum can yield sufficient DNA for methylation analyses. Only minor signs of degradation over time and differences with respect to pre-analytical sample handling were identified. The successful WGBS of ultralow DNA yields from archival (stored up to 48 years) serum samples shows the potential to utilize large serum collections for global methylation studies and biomarker discoveries.

Previous studies using archived serum have shown that it is possible to isolate DNA for genotyping and miRNA for biomarker studies [2, 3, 6]. These results, in conjunction with the present epigenetics study, indicate that it is possible to increase the potential for undertaking large-scale biomarker studies, combining several omics levels, in this biorepository.

## Methods

### Janus serum samples

The Janus Serum Bank at the Cancer Registry of Norway is a collection of serum samples from 318,628 Norwegian individuals, gathered between 1972 and 2004 [4]. Rich metadata are associated with most of these samples (i.e. age, smoking, body mass index). The samples have been stored up to 48 years at − 25 °C [4]. Pre-analytical treatments differed over the collection time and included treatments such as the addition of iodoacetate, varying clotting time, use of separating gel and lyophilized serum. For our pilot experiment, we selected 96 samples for DNA isolation that covered all treatments and collecting periods (blood donor groups 1–6; Table 1).

**Table 1:** Samples groups from the Janus Serum Bank repository

Group	Sample collection period	Sample source	Serum processing	Produced reads M range (mean)	Analysed Cs (M) range (mean)	Analysed for DNA yield (*N*)
1	1972–1978	HE	Iodoacetate added	287–889 (602)	3642–12,188 (8102)	10
2	1979–1986	HE	No additives	450–892 (707)	6200–11,843 (9586)	13
3	1987–2004	HE	Separating gel tubes	202–898 (644)	2573–12,184 (8650)	39
4	1973–1979	RCBD	Lyophilisation	376–889 (666)	5005–12,270 (8983)	14
5	1980–1990	RCBD	No additives	356–854 (626)	4611–11,850 (8341)	13
6	1997–2004	RCBD	No additives	327–685 (602)	4881–9480 (8059)	5
Total				94 out of 96		94 out of 96

The samples are collected as part of national health examinations (HE), or from red cross donors (RCBD). Pre-analytical handling differs between groups. The last columns show the number of reads produced (millions, range and mean indicated), number of C analysed (millions, range and mean indicated) and number of samples used for calculating DNA yield, respectively. Further description of the groups can be found somewhere else [2, 4, 6]

### Sample preparations

DNA was isolated manually from 500µL serum using the QIAamp DNA Blood Mini kit (Qiagen, Venlo, NL) and 0.03 mg of linear acrylamide (Invitrogen, AM9520). DNA concentration was measured using the Qubit™ dsDNA assay (Thermo Fisher Scientific). Extracted DNA underwent shearing treatment on Covaris LE220 instrument according to the manufacture’s recommendations (Massachusetts, USA) and using 1% trueSHEAR™ buffer (Covaris, Massachusetts, USA), followed by the bisulphite treatment performed using Zymo EZ-96 DNA Methylation-Lightning kit on a 96-well plate (D5033, Zymo Research, USA). We monitored the distribution of DNA size in samples throughout this process on TapeStation (Agilent, USA) according to recommendations from the manufacturer. All 96 samples with isolated DNA were subjected to Accel-NGS® Methyl-Seq DNA library preparation kit (Swift Biosciences, USA) with the adaptase module (Swift Biosciences, USA). The polymerase used in the extension reaction is a uracil-tolerant high-fidelity proofreading polymerase. The nine PCR amplification cycles were performed on 4–20 ng of DNA.

### Sequencing and bioinformatics processing

Sequencing was done on the NovaSeq 6000 sequencing platform, with S4 flow cell from Illumina (NGI, Sweden), using 100 bp paired-end reads and 5 samples per lane. The libraries were pooled equimolarly and sequenced within 1 month. The created libraries were sequenced in 5 batches. Prior to sequencing, the libraries were spiked with between 12 to 25% PhiX DNA to increase methylation library complexity and thus decrease potential problems with clustering during sequencing. Data pre-processing was done on secure SNIC/UPPMAX servers (NGI, Sweden) and Colossus servers (TSD, Norway). To pre-process the bisulphite sequencing data, we used ‘nf-core/methylseq’ v1.4 bioinformatics analysis pipeline, with the Accel-NGS parameter [10]. The complete information about the pipeline can be found in Additional file 1: Sup1. This pipeline used Bismark to generate a reference genome index [11], FastQC [12] to perform quality control of raw reads data [12] and TrimGalore! [13] to remove the adapters and trim low quality reads and leftovers from the adaptase technology. Read alignments, deduplications, the methylation calls extractions and sample reports were further done with Bismark [11]. Sample complexity and estimated library diversity was done with Preseq [14]. The Preseq software predicts the number of distinct reads that additional sequencing can be expected to produce given data from an initial sequencing experiment. These estimates can then be used to examine the utility of further sequencing, to optimize the sequencing depth or to screen multiple libraries to avoid low complexity samples. Finally, MultiQC was used to comprehensively present the project report [15]. To assess the origin of DNA fragments in serum samples sequenced in this project, we used the Houseman method [8] and methylCC [16] for comparison. We used Qualimap for general statistics as well as alignment, coverage and GC content [17]. To estimate the stability of the DNA, we measured the evenness of coverage distribution [5] and GC content. The evenness quantifies the homogeneity of coverage of the NGS targets and was calculated as the coefficient of variation for non-normalized data as previously described [5]. Changes in GC ratios would suggest DNA contamination, library preparations problems or DNA degradation. In statistical analysis, the storage time of the samples was treated as an ordered factor.

## Supplementary Information

**Additional file 1**. The detailed pipeline command used in the pre-processing analysis and workflow. SFig1: Percentage of methylation calls in data in blood donor groups.

## Data Availability

The datasets generated and analysed during the current study are not publicly available since individual privacy could be compromised, but are available from the corresponding authors on request and with appropriate approvals.

## Abbreviations

WGBS: Whole-genome bisulphite sequencing
NGS: Next generation sequencing
EWAS: Epigenome-wide association studies
GWAS: Genome-wide association studies
CpGs, or CG sites: Regions of DNA where a cytosine nucleotide is followed by a guanine nucleotide
PhiX: Bacteriophage single-stranded DNA

## References

[1] Li M, Zou D, Li Z, Gao R, Sang J, Zhang Y (2019). EWAS Atlas: a curated knowledgebase of epigenome-wide association studies. Nucleic Acids Res.

[2] Rounge TB, Lauritzen M, Erlandsen SE, Langseth H, Holmen OL, Gislefoss RE (2020). Ultralow amounts of DNA from long-term archived serum samples produce quality genotypes. Eur J Hum Genet.

[3] Groen K, Lea RA, Maltby VE, Scott RJ, Lechner-Scott J (2018). Letter to the editor: blood processing and sample storage have negligible effects on methylation. Clin Epigenet.

[4] Hjerkind KV, Gislefoss RE, Tretli S, Nystad W, Bjørge T, Engeland A, Meyer HE, Holvik K, Ursin G, Langseth H (2017). Cohort profile update: the Janus serum bank cohort in Norway. Int J Epidemiol.

[5] Oexle K (2016). Evaluation of the evenness score in next-generation sequencing. J Hum Genet.

[6] Rounge TB, Lauritzen M, Langseth H, Enerly E, Lyle R, Gislefoss RE (2015). microRNA biomarker discovery and high-throughput DNA sequencing are possible using long-term archived serum samples. Cancer Epidemiol Biomark Prev.

[7] Darst RP, Pardo CE, Ai L, Brown KD, Kladde MP (2010). Bisulfite sequencing of DNA. Curr Protoc Mol Biol.

[8] Houseman EA, Accomando WP, Koestler DC, Christensen BC, Marsit CJ, Nelson HH (2012). DNA methylation arrays as surrogate measures of cell mixture distribution. BMC Bioinform.

[9] Liu X, Ren J, Luo N, Guo H, Zheng Y, Li J (2019). Comprehensive DNA methylation analysis of tissue of origin of plasma cell-free DNA by methylated CpG tandem amplification and sequencing (MCTA-Seq). Clin Epigenet.

[10] Ewels PA, Peltzer A, Fillinger S, Patel H, Alneberg J, Wilm A (2020). The nf-core framework for community-curated bioinformatics pipelines. Nat Biotechnol.

[11] Krueger F, Andrews SR (2011). Bismark: a flexible aligner and methylation caller for Bisulfite-Seq applications. Bioinformatics.

[12] Babraham bioinformatics—FastQC a quality control tool for high throughput sequence data. [Cited 2021 Mar 18]. https://www.bioinformatics.babraham.ac.uk/projects/fastqc/.

[13] FelixKrueger. FelixKrueger/TrimGalore [Internet] [Cited 2021 Mar 18]. https://github.com/FelixKrueger/TrimGalore.

[14] Daley T, Smith AD (2013). Predicting the molecular complexity of sequencing libraries. Nat Methods.

[15] Ewels P, Magnusson M, Lundin S, Käller M (2016). MultiQC: summarize analysis results for multiple tools and samples in a single report. Bioinformatics.

[16] Hicks SC, Irizarry RA (2019). methylCC: technology-independent estimation of cell type composition using differentially methylated regions. Genome Biol.

[17] García-Alcalde F, Okonechnikov K, Carbonell J, Cruz LM, Götz S, Tarazona S (2012). Qualimap: evaluating next-generation sequencing alignment data. Bioinformatics.

