# Supplementary material for: Ultralow amounts of DNA from long-term archived serum samples produce high-quality methylomes
Source: Clin Epigenetics. 2021 May 12;13:107. doi: 10.1186/s13148-021-01097-3 (PMC8114536; doi:10.1186/s13148-021-01097-3)

**Supplementary online material** for ‘Ultralow amounts of DNA from long-term archived serum samples produce high-quality methylomes’ by Marcin W. Wojewodzic* , Magnus Leithaug , Marianne Lauritzen , Robert Lyle , Sofia Haglund , Carl-Johan Rubin, Philip A. Ewels, Tom Grotmol, Trine B. Rounge*

**Supplementary 1** The detailed pipeline command used in the preprocessing analysis can be found under the following GitHub repository: <https://github.com/nf-core/methylseq> where following tools were used: nf-core/methylseq v1.4, Nextflow v19.04.1, Bismark genomePrep v0.22.2, FastQC v0.11.8, Cutadapt v1.18, Trime Galore! V0.6.4, Bismark v0.22.2, Samtools v1.9, Picard MarkDuplicates v1.4, Preseq v2.0.3, MultQC v1.7

## Workflow summary of the methylseq pipeline contained following arguments: --reads data/*_R{1,2}.fastq.gz, --aligner bismark, --fastaRef ./Homo_sapiens.GRCh38 .dna_sm.primary_assembly.fa --TrimProfile Accel-NGS (Swift), --Trim R1 10, --Trim R2 15, --Trim 3’R1 10, --Trim 3’R2 10, --Duplications Yes, --Directional Mode Yes.

## Workflow was run in the singularity container methylseq.1.4.simg with maximal resources 250 GB memory, 16 cpu, 10 days per job given.

**Supplementary SFig1** Percentage of methylation calls in data in blood donor groups for A) Cytosines in CpG context and B) for Cytosines in non-CpG context (here CHH)


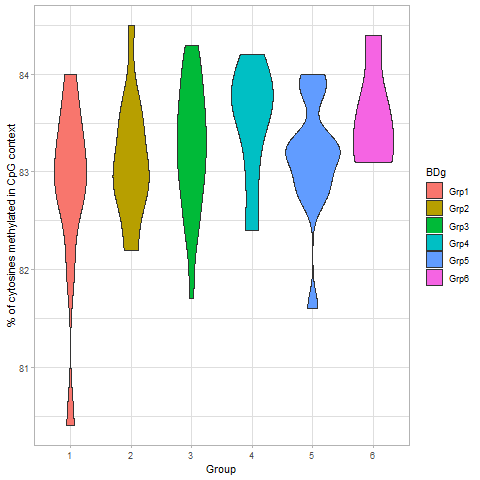

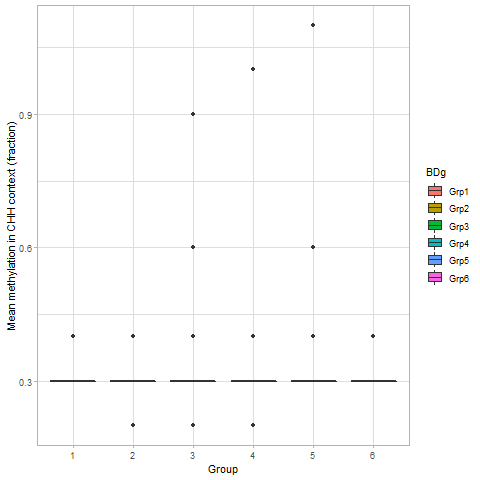

Supplement: Supplementary file 1 — Additional file 1. The detailed pipeline command used in the pre-processing analysis and workflow. SFig1: Percentage of methylation calls in data in blood donor groups. [file 13148_2021_1097_MOESM1_ESM.docx]
